# Transcutaneous Vagus Nerve Stimulation During Motor Activity in Healthy Volunteers: A High-Density Diffuse Optical Tomography Study

**DOI:** 10.3390/brainsci16020146

**Published:** 2026-01-29

**Authors:** Sheharyar S. Baig, Caitlin H. Illingworth, Breanna McQueen, Amy Gibbons, Joanna Ravenscroft, Charlotte Morton, Gavin Brittain, Emilia Butters, Sabrina Di Lonardo Burr, Ali N. Ali, Arshad Majid, Li Su

**Affiliations:** 1 Sheffield Institute for Translational Neuroscience, School of Medicine and Population Health, University of Sheffield, Sheffield S10 2HQ, UK; 2 School of Psychology, University of Sheffield, Sheffield S1 4DP, UK; 3 Department of Electrical Engineering, University of Cambridge, Cambridge CB3 0FA, UK; 4 Department of Psychiatry, School of Clinical Medicine, University of Cambridge, Cambridge CB2 0SZ, UK

**Keywords:** vagus nerve stimulation, functional near-infrared spectroscopy, diffuse optical tomography, non-invasive brain stimulation, rehabilitation, stroke

## Abstract

**Background:** Stroke is a leading cause of long-term disability worldwide. Non-invasive or transcutaneous auricular vagus nerve stimulation (taVNS) shows promise in promoting neuroplasticity and supporting motor recovery. There are currently no validated biomarkers of taVNS. High-density diffuse optical tomography (HD-DOT) is a portable neuroimaging technology that uses near-infrared light to map cortical activity via the quantification of changes in blood oxygenation. The aim of this study was to determine whether HD-DOT could detect motor task-related activity with concurrent taVNS. **Methods:** Thirty-one healthy participants completed right and left finger tapping tasks with concurrent sham (earlobe) and then active (tragus) taVNS in a within-subject block design. HD-DOT was recorded across the bilateral sensorimotor cortex using 36 sources and 48 detectors (1728 channels). Cortical reconstructions were parcellated and block-averaged task-related oxygenated and deoxygenated haemoglobin changes were compared between sham and active taVNS conditions. **Results:** In a group-level analysis, appropriate lateralised task-related haemodynamic responses were seen in the contralateral sensorimotor regions, demonstrating the validity of HD-DOT. Between-group comparisons showed no significant change in task-related activation during right finger tapping tasks under active vs. sham taVNS conditions. A non-significant redistribution of task-related activity to the right motor cortex was seen with left finger tapping under active taVNS compared to sham taVNS. **Conclusions:** Simultaneous recording of neural responses to taVNS during motor activity was feasible and well tolerated. Reliable task-related activation was recordable. Future studies of whole brain HD-DOT in people with stroke will help evaluate its potential as a biomarker in taVNS.

## 1. Introduction

Stroke is a leading cause of adult-onset disability affecting 12.2 million people each year [1]. Approximately 50% of people have persistent arm weakness which significantly impacts their ability to perform daily tasks and their quality of life [2]. Whilst rehabilitation has been shown to promote motor plasticity in chronic stroke (>6 months post-onset), the magnitude of improvement is small [3] and the number of hours of rehabilitation required is cost-prohibitive in many settings [4]. As such, adjuncts to motor rehabilitation that synergise with physiotherapy are an area of unmet need and a priority for stroke survivors.

Invasive vagus nerve stimulation (VNS) synchronised with motor rehabilitation has been shown to improve arm motor recovery in chronic ischaemic stroke [5] and is the only U.S. Food and Drug Administration (FDA)-approved adjunct to motor rehabilitation. In a pivotal multicentre VNS-REHAB study, 6 weeks of invasive VNS delivered alongside upper limb rehabilitation led to a clinically meaningful improvement in upper limb function (Fugl Meyer Upper Extremity total motor score increase of ≥6 points) in 47% of participants in the active VNS group vs. 24% of participants in the sham VNS group. VNS is thought to increase motor plasticity through cholinergic reinforcement of motor learning [6] and to influence other modulatory neurotransmitters such as noradrenaline, serotonin, dopamine and gamma-aminobutyric acid (GABA) [7]. Non-invasive or transcutaneous vagus nerve stimulation (tVNS) refers to the delivery of VNS through the skin via either the auricular branch in the ear (taVNS) or the cervical branch in the neck (tcVNS) [8]. TaVNS has several advantages over invasive VNS. It is not associated with any serious adverse events, does not require surgical implantation and can be used immediately after stroke [8]. Pilot and feasibility studies of taVNS after stroke are promising, showing increases in upper limb motor function in individuals with subacute and chronic stroke [9,10] and the efficacy of taVNS in stroke is currently being assessed in several multicentre randomised controlled trials [7,11].

There are several candidate biomarkers of taVNS-related activation including heart rate variability, P300 potential, and salivary alpha amylase [12]. However, none have reliably been shown to consistently detect activation-related changes in healthy volunteers [12]. Whilst functional magnetic resonance imaging (MRI) has shown taVNS-related activation in the visuomotor areas and secondary visual cortex [13], it is not known how taVNS-related activation is modulated during motor tasks. Identifying biomarkers of the taVNS–movement interaction would enable more efficient clinical trial design and potentially individualise stimulation parameters (laterality, amplitude, frequency, and pulse width) by demonstrating engagement with recovering sensorimotor areas.

Functional near-infrared spectroscopy (fNIRS) uses near-infrared light to monitor the haemodynamic response to cerebral activity [14]. Increases in neural activity are associated with an increase in local oxygenated blood to support this activity which can be detected via increases in oxygenated haemoglobin (HbO) and decreases in deoxygenated haemoglobin (HbR) [14]. Traditional fNIRS systems have been low-density (16–64 channels). High-density diffuse optical tomography (HD-DOT) uses fNIRS with dense optode arrays and structural priors that allow the recovery of 3D images of oxygenation changes, which yield much higher spatial resolution than low-density fNIRS [15,16].

No prior studies have utilised HD-DOT to monitor the effects of VNS. The aim of the present study is to determine whether taVNS modulates cortical haemodynamic responses during a simple motor task, using HD-DOT in healthy adults.

## 2. Materials and Methods

### 2.1. Participants

A total of 31 healthy adults (20 female, 11 male) were recruited through convenience sampling from the University of Sheffield. Given our recruitment strategy, the cohort was primarily young adults (*M_age_* = 23.4, *SD* = 2.9). Participants were excluded if they had contraindications to taVNS use as per the Nurosym^TM^ device manual:

- Had open wounds affecting the scalp or ears.
- Were pregnant.
- Had a prior vagotomy.
- Had non-removable piercings at stimulation sites (i.e., left tragus or earlobe)
- Had implanted electronic medical devices such as pacemakers or cochlear implants.
- Had a known history of symptomatic bradycardia, second- or third-degree atrioventricular block, or carotid artery stenosis exceeding 50%.

### 2.2. Experimental Design

A within-subjects block design was employed to investigate the cortical effects of taVNS on motor execution and motor imagery tasks. Following informed consent, each participant completed a demographic form recording age, sex, ethnicity, handedness, hair colour and hair length. Each participant completed six experimental conditions:

1. Right-hand finger tapping (no stimulation);
2. Right-hand motor imagery (no stimulation);
3. Right-hand finger tapping with sham taVNS (left earlobe);
4. Left-hand finger tapping with sham taVNS (left earlobe);
5. Right-hand finger tapping with active taVNS (left tragus);
6. Left-hand finger tapping with active taVNS (left tragus).

**Figure 1:**
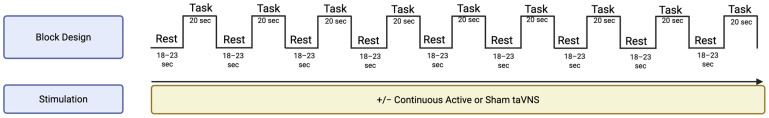
Illustrative Block Design for one experimental condition.

For each condition, the experimental paradigm was structured in nine discrete blocks, each consisting of a 20 s task period followed by a pseudo-jittered rest period (18–23 s) (Figure 1). During the task, participants either performed self-paced, unilateral finger tapping at approximately 1 Hz or imagined finger tapping with their right hand (motor imagery task). Participants were trained in the finger tapping rate before the experimental recording and consistency in tapping rate and force was monitored. The experimental conditions were counterbalanced for left and right finger tapping and presented in Order A (1, 2, 3, 4, 5, 6) or Order B (2, 1, 3, 4, 6, 5), with an alternating sequence for consecutive participants. The data for the right-hand finger tapping task without stimulation and motor imagery task (1 and 2) are the focus of a separate study on motor imagery.

### 2.3. Experimental Set-Up

**Figure 2:**
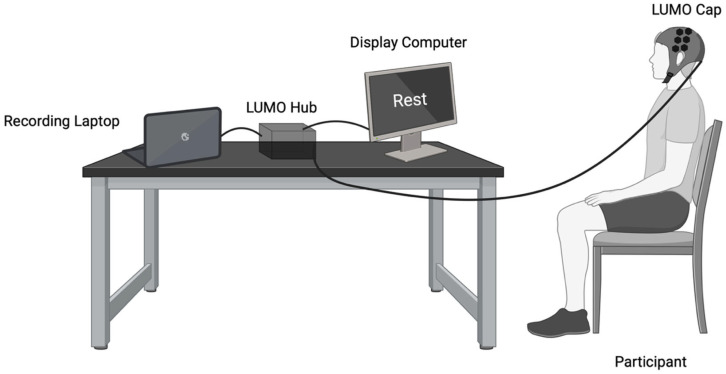
The experimental set-up included the HD-DOT acquisition laptop (left), the LUMO HD-DOT hub (middle, black box) and a separate experimental presentation computer (right). The participant wears a neoprene cap with 12 hexagonal tiles (36 sources, 48 detectors), placed over the sensorimotor cortex.

Visual written cues indicating the current block type (“Rest” or “Finger Tapping”) were presented using PsychoPy (v2024.2.24) on a 14-inch laptop positioned approximately 60 cm from the participant for the duration of the task. Prior to the start of the experiment, researchers provided verbal instructions and demonstrated the tapping procedure. Data collection took place in a private room at the University of Sheffield or in the Royal Hallamshire Hospital Biomedical Research Centre. The experimental set-up is illustrated in Figure 2.

### 2.4. HD-DOT Acquisition

**Figure 3:**
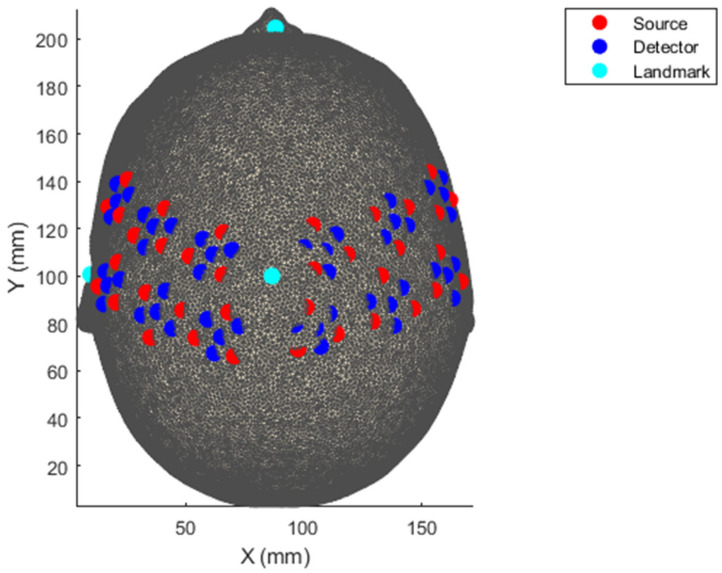
Source–Detector Registration to template brain showing locations of optodes and cranial landmarks using the assumed locations provided by the HD-DOT System.

The data were collected using the 56–58 cm LUMO system (Gowerlabs Ltd., London, UK), a high-density continuous wave fNIRS system. Optical data were acquired using 12 tiles placed over the bilateral sensorimotor cortex, which sampled at 12.5 Hz (Figure 3). The LUMO system consists of multidistance overlapping channels, enabling the dissociation of haemodynamic data from the scalp (10–12 mm channels) and the cortex (12–42.5 mm) [17]. Each tile consists of three dual-wavelength LED sources (735|850 nm) and four photodiode detectors, for a total of 36 sources and 48 detectors, where each source forms a channel with each detector, for a total of 1728 possible channels. Default optode locations were used for all subsequent analyses as no digitisation was conducted to register the location for each participant.

### 2.5. TaVNS Delivery

**Figure 4:**
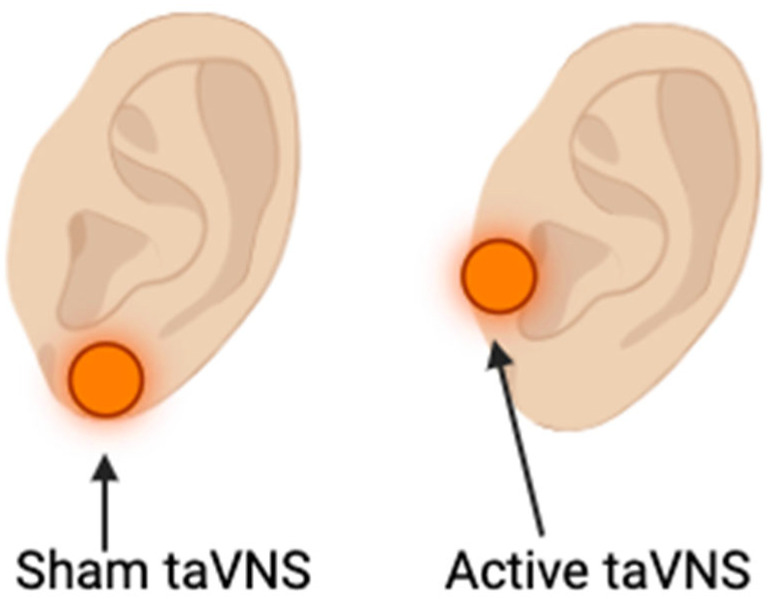
Stimulation sites for sham and active taVNS. (**left**) Sham stimulation was delivered at the left earlobe. (**right**) Active stimulation was delivered at the left tragus.

TaVNS was delivered using the Nurosym^TM^ device (Nurosym Ltd., London, UK). During active stimulation, electrodes were placed on the left tragus, a site innervated by the auricular branch of the vagus nerve [17] (Figure 4). For sham stimulation, electrodes were positioned on the left earlobe, which lacks vagal innervation and is widely used as a control site in taVNS studies [18].

In both active and sham conditions, stimulation was delivered continuously throughout each task block at a fixed frequency (25 Hz) and pulse width (250 μs), consistent with prior taVNS protocols [8].

### 2.6. HD-DOT Preprocessing

Data preprocessing was conducted using MATLAB R2023b (MathWorks, Natick, MA, USA), with a custom pipeline that incorporated functions from Homer2 [19] and the DOT-HUB toolboxes (https://github.com/DOT-HUB (accessed on 15 December 2025)).

Raw light intensity signals were first converted to changes in optical density (ΔOD) using the Homer2 toolbox [19]. Channels were excluded if the raw light intensity was outside [0, 1 × 10^11^], the signal-to-noise ratio fell below 12, or source–detector distances were over 100 mm [20,21]. Motion artefact burden was derived using the DOTHUB_dataQualityCheck, which found low motion burden across the recordings (Median = 0%, *M* = 20.5%, SD = 38.3%). Given the low prevalence of motion artefacts in this task, no motion artefact correction was applied. Optical density data was converted to concentration changes in HbO and HbR using the hmrOD2Conc function with a differential pathlength factor of 6. Short-channel regression (10–12 mm) was applied using the DOTHUB_hmrSSRegressionByChannel function to reduce superficial signal contamination, followed by a third-order Butterworth bandpass filter (0.01–0.1 Hz) [22]. In the instance of missing values due to system error lasting less than 1 s (8 recordings: 2 Active Left Motor, 1 Sham Left Motor, 4 Active Right Motor, 1 Sham Right Motor), data was imputed using the mean of the two neighbouring values. Task-evoked haemodynamic responses were extracted using block averaging, time-locked to stimulus onset. Averaging windows spanned −2 to +20 s, with −2 to 0 s defined as the baseline and 10–20 s post stimulus onset as the task period for analysis. The time window of 10–20 s for the task-related activity was chosen to capture the peak, stable plateau of task-related activity, accounting for the delay in haemodynamic response from task-onset.

### 2.7. Participant Exclusion

All participants completed the full experimental protocol. Recordings were excluded from further analysis if <1/3 of channels within 10–42.5 mm met pruning criteria based on the work by Fiske et al. (2022) [23]. This resulted in the exclusion of 11–14 participants across the conditions for the node-level analysis (see Table 1).

### 2.8. Image Reconstruction and Parcellation

For each participant, a map of cortical oxygenation was reconstructed using a tetrahedral grey matter mesh based on the standard MNI-152 template head model [24]. For reconstruction, concentration changes were converted to optical densities using DOTHUB_hmrConc2OD. To reconstruct the optical data, a forward model of light propagation was calculated using the DOTHUB_make_Jacobian function. From this, a zeroth-order Tikhonov regularised inversion was performed using a regularisation hyperparameter of 0.01.

**Figure 5:**
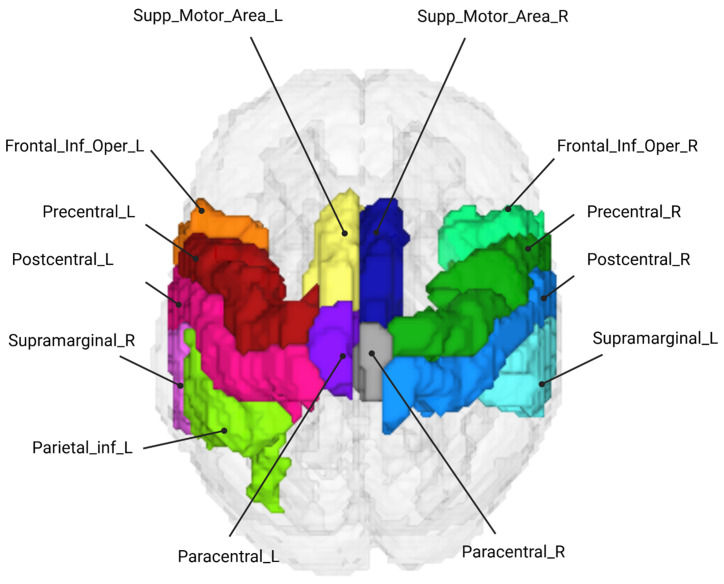
Cortical regions from the AAL2 atlas targeted in this study using the HD-DOT motor cap. These parcels defined the 13 motor ROIs for group-level parcellation analysis.

**Figure 6:**
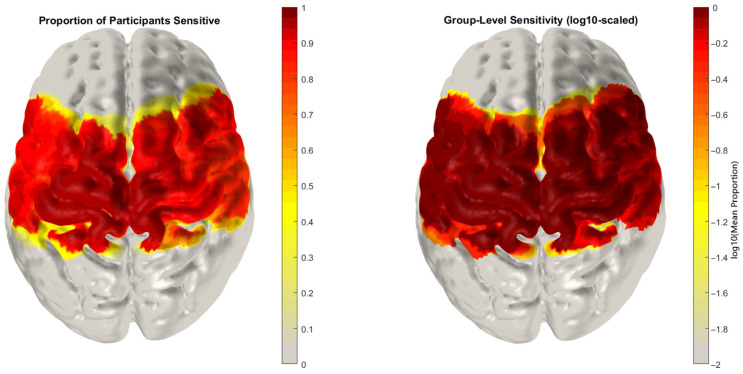
Average sensitivity of motor array nodes across participants (**left**). Proportion of participants with nodes sensitive to HbO and HbR changes across regions of the cortex (**right**) averaged group level sensitivity to HbO and HbR changes. Node sensitivity is defined using the thresholds proposed by Uchitel et al. (2022) [26] for detecting changes in HbO and HbR.

**Figure 7:**
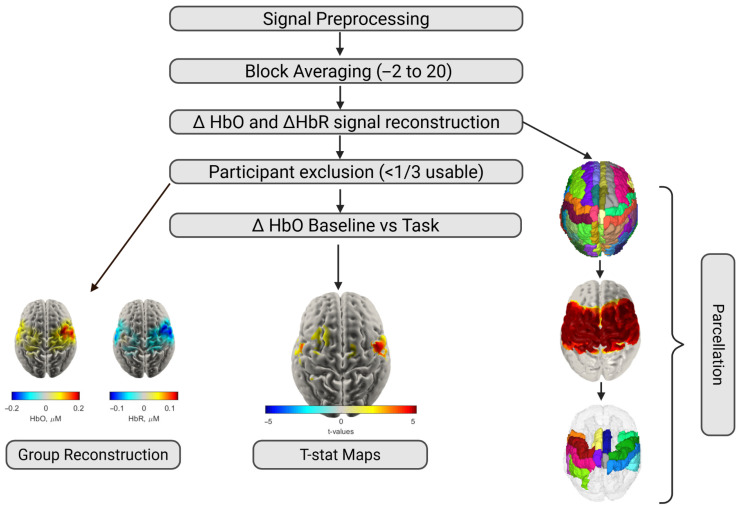
Overview of analysis pipeline from raw infrared light signal to 3D reconstruction and statistical testing.

Reconstructed data for each participant were then parcellated using the AAL2 Atlas [25], assigning each grey matter mesh node to one of thirteen corresponding motor-related regions of interest (ROIs) (see Figure 5). A node in the grey matter mesh was defined as sensitive if its sensitivity in the Jacobian matrix exceeded 5% of the maximum value of the normalised Jacobian for both wavelengths (Figure 6) [26]. For each participant, parcels were only included in subsequent averaging and analysis if over 50% of the nodes within them were sensitive to both HbO and HbR changes. A single time series was then calculated for each parcel by averaging the time series of all sensitive nodes within that parcel. Only parcels for which two or more participants were sensitive across >50% of nodes within the parcel were included in the statistical analysis. This resulted in a total of 13 parcels included in the statistical analysis. The imaging analysis pipeline is outlined in Figure 7.

### 2.9. Statistical Analysis

For each parcel, task-evoked changes in HbO and HbR were calculated based on baseline and task windows. ΔHbO is presented in the main analysis due to its higher signal-to-noise ratio [27]. For each participant, an average time series of HbO and HbR concentrations was derived from the nodes within the parcel. From this, mean HbO concentrations were extracted for the baseline (−2 s to 0 s) and chosen window of activation during the task period (10–20 s post-stimulus) for each parcel. Looking at within-condition changes, paired *t*-tests were conducted for all parcels between the group average baseline and task window mean HbO. When comparing between conditions, the mean ΔHbO from baseline to task was calculated for each parcel and each condition. These calculated ΔHbO values were compared using a paired *t*-test for each parcel. Multiple comparisons were corrected using the false discovery rate (FDR) method [27]. Both corrected and uncorrected *p*-values are reported.

## 3. Results

### 3.1. Participant Characteristics

**Figure 8:**
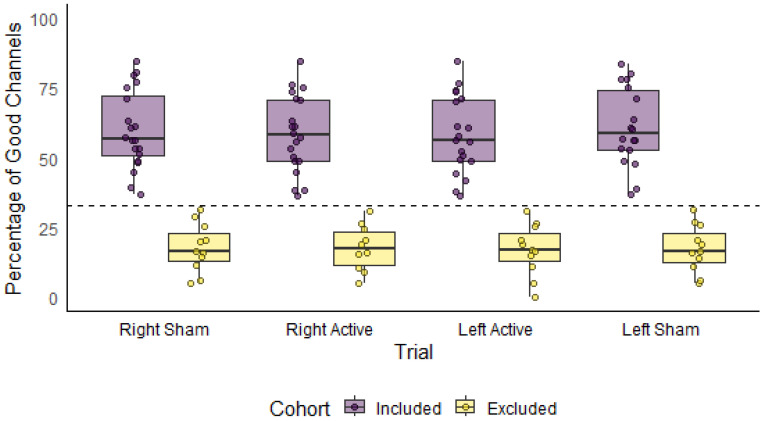
Percentage of ‘good’ channels within each task for each participant, grouped by inclusion status. Note that the total possible number of channels within the 10–42.5 mm range is 464. The horizontal dashed line represents the minimum 33.3% (1/3) channel threshold for including a recording.

All participants completed the conditions within the same session and each condition was saved as a separate recording. Across condition recordings, the number of channels that met the pruning criteria varied, leading to some participants being excluded from some, but not all conditions. The characteristics for each cohort used in the group-level analyses are reported in Table 1. Figure 8 illustrates the percentage of good quality channels by condition, stratified by included and excluded participants. After exclusion, the average number of good channels across participants did not significantly differ between conditions (F(3) = 0.18, *p* = 0.91) (see Supplementary Table S1).

### 3.2. taVNS Parameters

Participants generally tolerated taVNS well across sham and active sessions, with no sessions stopping due to participant discomfort. The taVNS intensity did not significantly differ between active and sham trials (*t*(30) = 1.05, *p* = 0.30). Mean stimulation amplitudes are shown in Table 2.

### 3.3. Group-Average Maps

To visualise the cortical distribution of task-evoked responses, group-level HbO activation maps were generated for right-hand finger tapping execution under both sham and active taVNS conditions. These maps represent the average HbO and HbR concentrations during the task period, 10–20 s post-stimulus onset.

**Figure 9:**
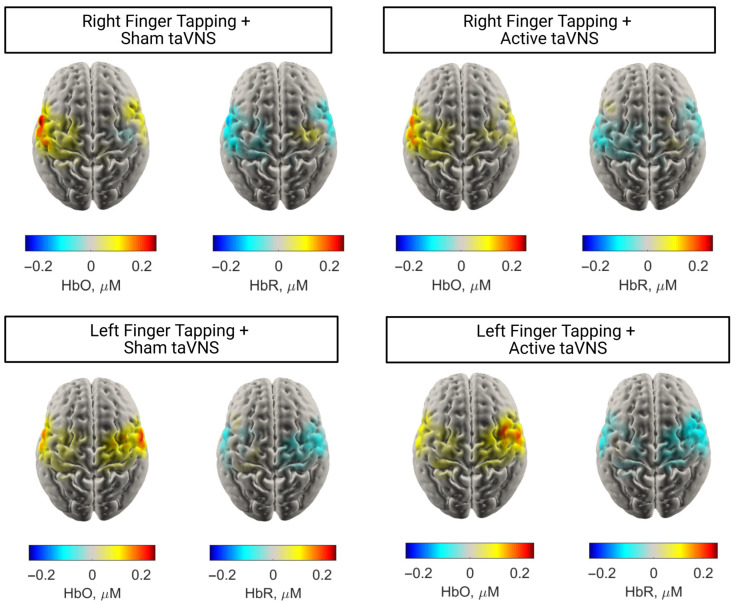
Cortex surface reconstructions of the average reconstructed HbO and HbR concentrations across all participants with >⅓ good channels.

As would be expected, in both right motor active and sham conditions (Figure 9), similar patterns of HbO concentration changes were observed, with the greatest concentration change in the left (contralateral) motor cortex. There was also evidence of a coincident decrease in HbR concentration in the same region. In contrast, during the left-sided motor tasks, there appears to be greater activation in the ipsilateral hemisphere during the sham condition compared to the active taVNS condition.

### 3.4. Statistical Analysis of taVNS on HD-DOT

**Figure 10:**
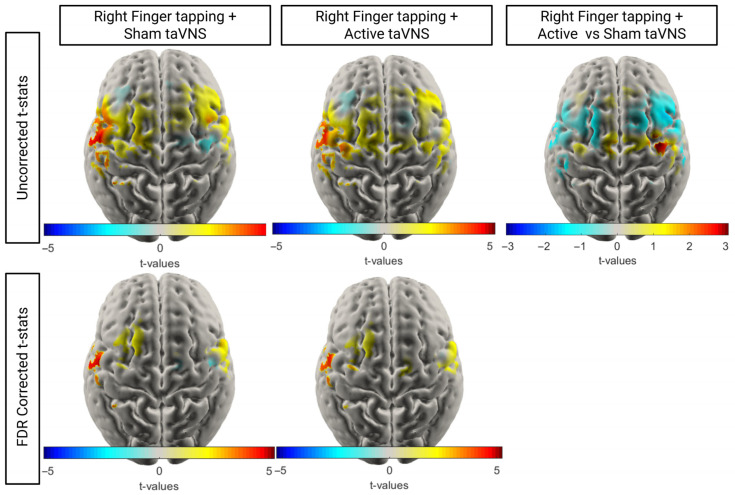
T-stat maps comparing HbO at baseline versus task within conditions and relative change in HbO concentration across right finger tapping tasks (*n* = 20). For the uncorrected t-maps, only nodes with a *p* < 0.05 prior to FDR correction are shown.

**Figure 11:**
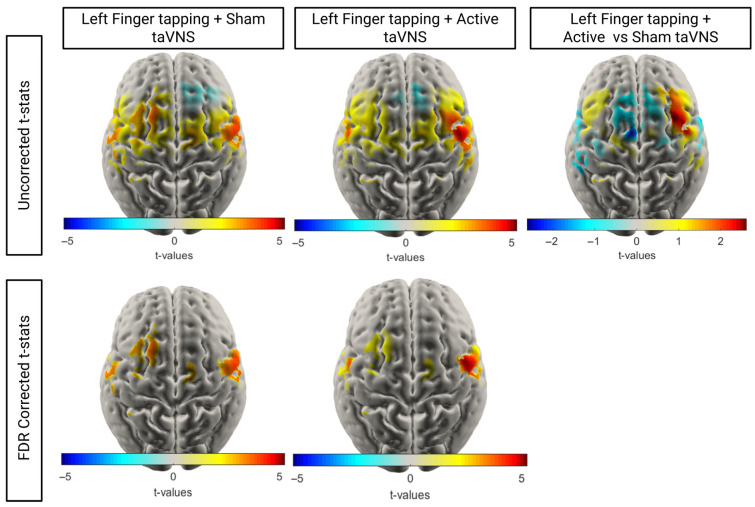
T-stat maps comparing HbO at baseline vs. task within conditions and relative change in HbO concentration across left finger tapping tasks (*n* = 17). For the uncorrected t-maps, only nodes with a *p* < 0.05 prior to FDR correction are shown.

To investigate the effect of taVNS on cortical activation, we conducted *t*-tests within tasks to compare HbO concentration at baseline versus during the task for each node. To identify significant differences in task-related activation across conditions, node-wise paired *t*-tests were performed between the mean ΔHbO (HbO task window (10–20 s) − HbO (baseline −2 to 0 s)) for each node in one condition compared to another. Data was matched between conditions, with only individuals who had over ⅓ usable channels for both conditions included in the *t*-tests. Corrected and uncorrected t-stat maps are shown in Figure 10 and Figure 11. Equivalent HbR maps are shown in the Supplementary Material (see Supplementary Figures S1 and S2).

As with the average concentration maps, the active and sham right motor taVNS t-stat maps exhibited very similar typical patterns of activation, with the greatest activation seen in the left motor regions. When comparing the activation seen across the active and sham conditions (*n* = 20), we observed a slightly greater increase in HbO in the right posterior motor regions during active stimulation. However, this effect did not survive FDR correction.

During the left motor tasks, participants exhibited more bilateral activation in the motor regions when receiving sham stimulation. In contrast, participants exhibited a more exclusively contralateral HbO increase when receiving active stimulation. Direct comparison of the calculated HbO change seen in active compared to sham (*n* = 17) reflects this, with greater HbO concentration increases seen in the right hemisphere and less activation in the left hemisphere. However, these differences did not survive FDR correction, indicating that if there is a neuromodulatory effect of taVNS on activation, it may have been too small for us to detect in the current study.

### 3.5. Parcellation Analysis

To compare the activation seen across stimulation conditions, paired *t*-tests were conducted using the mean ΔHbO across all nodes from within each parcel from the defined baseline (−2 to 0 s) to the active period (10–20 s). When comparing the mean ΔHbO across parcels between stimulation conditions, i.e., *t*-test: ΔHbO (active) vs. ΔHbO (sham), no contrasts were significant, even without FDR correction (see Supplementary Material). Within-task *t*-tests were conducted between the mean HbO at baseline compared to during the task. Equivalent HbR comparisons are shown in the Supplementary Material (see Supplementary Tables S3, S5–S7).

During right motor tasks, several regions in the somatosensory motor area in the left hemisphere showed a significant increase in HbO concentration (see Table 3). When receiving active taVNS, fewer regions exhibited a significant increase in HbO concentration compared to when receiving sham taVNS, namely just the left pre- and post-central gyri. However, when comparing the changes in HbO concentrations across stimulation conditions (*n* = 20), no contrasts were significant, even without FDR correction (see Supplementary Table S2).

During left motor tasks, several regions in the somatosensory motor area in the left hemisphere showed a significant increase in HbO concentration (see Table 4). As with the right motor tasks, when receiving active taVNS, fewer regions exhibited a significant increase in HbO concentration compared to when receiving sham taVNS. However, when comparing the changes in HbO concentrations across stimulation conditions (*n* = 17), no contrasts were significant, even without FDR correction (see Supplementary Table S2).

## 4. Discussion

This is the first study to use HD-DOT to study the effects of taVNS. Recording the effects of taVNS with concurrent motor tasks using HD-DOT was feasible and well-tolerated. Task-related activity from unilateral finger tapping reliably activated contralateral sensorimotor areas in keeping with expected activation patterns [28]. Whilst no significant differences were observed between active and sham taVNS, this study provides foundational knowledge for future studies of neuromodulation and HD-DOT.

VNS has been postulated to activate the nucleus tractus solitarius (NTS) with further projections to the nucleus basalis (cholinergic), locus coeruleus (noradrenergic), ventral tegmental area (dopaminergic) and dorsal raphe nucleus (serotonergic) [8]. Prior studies of taVNS delivered immediately before functional MRI have shown increases in several brain regions [13]. For instance, Frangos et al. (2015) demonstrated activation of the nucleus tractus solitarius (NTS), the principal relay centre for vagal afferent input [18]. Concurrent invasive or taVNS with functional MRI is challenging due to MR compatibility issues. Peng et al. (2023) combined taVNS during functional MRI in stroke survivors and found that ipsilesional taVNS increased activation in ipsilesional visuomotor regions and decreased activation in contralesional visuomotor regions [29]. Despite the paradigm for taVNS-related rehabilitation combining stimulation with arm movement, no functional MRI studies have delivered taVNS alongside movement tasks.

Wang et al. performed a low-density fNIRS study in people with stroke where motor-task-related fNIRS was recorded immediately prior to and after a single stimulation session of taVNS [30]. This demonstrated that taVNS enhanced activation in the unaffected hemisphere premotor cortex/supplementary motor area and left Broca’s area in people with left hemiplegia and increased activation in the primary somatosensory cortex in those with right-sided hemiplegia. This suggests a neuromodulatory effect that does not directly enhance primary motor cortex activity. However, low-density fNIRS systems have limited spatial resolution and less capacity to control for physiological noise. Furthermore, as taVNS was not delivered in synchrony with movement, the shorter-acting neuromodulatory effects cannot be inferred. A recent study of three weeks of left cymba concha taVNS paired with task-orientated training of the upper limb in 30 individuals with subacute stroke found that several sessions of taVNS increases activity in the ipsilesional primary motor cortex, premotor area and supplementary motor area when performed under low cognitive load [31].

The current study explores a clinically relevant, ecologically valid setting where taVNS was delivered alongside upper limb movement. Group analysis took place at the node (voxel) level and at a regional level. Node-level analysis can provide granular detail at small foci of activation but can be susceptible to small between-condition variances and correction for multiple comparisons can lead to overcorrection. The parcellated region-of-interest analysis can reduce the number of comparisons by aggregating nodes within a region; this gives more easily interpretable changes in activation but can dilute the overall effect (Type II error) if only a subregion of a parcel changes or a cluster of activation crosses atlas boundaries.

At the parcel-level, task-related activity increased in the precentral gyri during active and sham motor tasks, even when corrected for multiple comparisons. This gives reassurance that task-related activity is reliably detected by HD-DOT using the current task paradigm and is one of the first demonstrations of HD-DOT in studies of motor function. At the node-level, right-sided finger tapping performed alongside sham stimulation led to expected activation in left sensorimotor areas, whilst corresponding left-sided finger tapping showed right-predominant but more bilateral representation. This may partly be explained by most participants being right-handed and prior studies suggesting that non-dominant hand representation has greater bilateral distribution than the dominant hand [32].

Active taVNS did not significantly change node-level or parcel-wide activation in either the right or left finger tapping tasks. However, at the node-level, the t-statistic maps indicated a redistribution of task-related activity in the left finger tapping task towards the right motor cortex. In the uncorrected between-condition comparison for active taVNS vs. sham taVNS during the left finger tapping task, a cluster of increased activation was seen in the right motor cortex. It is therefore possible that active taVNS paired with unilateral movement may have a lateralised effect in the contralateral hemisphere to stimulation that the current study is underpowered to detect. An alternative explanation is that, as the majority of the cohort were right handed, the left handed finger tapping is a less practiced task with potential for enhancement with neuromodulation whilst right finger tapping has an optimised haemodynamic response. This has important parallels in stroke rehabilitation research during which the motor tasks will have higher cognitive demand.

The underlying physiological basis for the potentially enhanced BOLD response seen is not known. It is possible that it could relate to increased neural activity from the modulatory effects of cholinergic and noradrenergic input into the motor cortex. Alternatively, autonomic regulation of cerebrovascular reactivity and cerebral blood flow may enhance local blood flow indirectly of the neural response. Future studies incorporating electrophysiological responses with HD-DOT could explore this question.

There are several clinical implications for the current study. If the effects of taVNS paired with movement preferentially enhance the contralateral motor cortex, then there would be a strong case for the delivery of stimulation contralateral to the lesioned hemisphere or bilaterally. Furthermore, HD-DOT can potentially demonstrate individual task-related activation in healthy volunteers. The optimal treatment parameters for VNS in clinical practice are not known and likely to be individual. HD-DOT could be used to individualise stimulation delivery in future studies of stroke survivors undergoing invasive or non-invasive VNS; for instance, current amplitude, frequency and pulse width could be modulated on an individual basis to target the greatest increase in task-related activation from stimulation. HD-DOT could be combined with other putative biomarkers, e.g., pupillometry and heart rate variability, to demonstrate whether the afferent vagus nerve is being reliably activated and stratify potential responders vs. non-responders.

### Strengths and Limitations

Some of the strengths of the current study are the novel use of a high-density motor array, the use of short-separation channels to reduce the influence of systemic increases in blood flow, the placebo-control for stimulation and the counterbalancing of left and right motor tasks. The reciprocal increases and decreases between HbO and HbR maps provide physiological validation of the HD-DOT measurements.

There are several limitations to the current study. First, the HD-DOT system used a dense motor cortex array; therefore, changes in neural activity within frontal or posterior regions could not be captured. Second, near-infrared light does not penetrate beyond approximately 2 cm into the cortex and therefore cannot be used to assess changes in subcortical activation. Third, lack of subject-specific registration means that we could not precisely localise task-related activation. The spatial uncertainty of localization in lateral motor regions is approximately 27.4 mm [33]. This limitation is minimised by the within-subject design, standardisation in cap placement and the parcellation approach which includes large anatomical parcels. Fourth, several participants had dark hair which may influence signal quality from source–detector pairing. Fifth, due to the uncertainties around the duration of taVNS-related neuromodulation (i.e., the washout period) and the potential for a carryover effect, the active stimulation conditions always followed the sham stimulation conditions which may have given rise to an order effect from habituation, attentional and physiological drift. This decision in choice of study design was taken deliberately, as recording within a single session ensures consistency in cap placement and is reflective of real-world interactions with patients where set-up occurs in a single visit. Sixth, determining the laterality of stimulation could also be assessed through right-sided taVNS; however, most commercial taVNS devices restrict recommended use to the left auricular branch. This is a carryover from studies of cervical stimulation where right cervical vagus nerve stimulation is avoided due to efferent cardiac innervation; future studies should explore right sided and bilateral auricular stimulation which have been demonstrated to be safe [34]. Seventh, whilst participants were trained to have a consistent finger tapping rate and amplitude, this was visually inspected and not formally quantified with an accelerometer or electromyography.

## 5. Conclusions

This study provides the first evidence of the feasibility of taVNS with HD-DOT and the first use of functional neuroimaging with synchronous taVNS and motor tasks. HD-DOT could potentially be used to track individual-level responses to taVNS and in the development of precision neuromodulation strategies. Future studies should aim to study both whole brain HD-DOT and real-time neurofeedback and to systematically test different stimulation parameters in patient cohorts.

## Figures and Tables

**Table 1:** Participant characteristics.

Characteristic	Total Cohort	Cohort After Quality Exclusion			
		Sham Right Motor	Active Right Motor	Sham Left Motor	Active Left Motor
	*n* (%) or *M* (*SD*)	*n* (%) or *M* (*SD*)	*n* (%) or *M* (*SD*)	*n* (%) or *M* (*SD*)	*n* (%) or *M* (*SD*)
*n*	31	20	20	18	19
Age (years)	23.42 (2.9)	23.1 (3.2)	23.1 (3.2)	23.2 (3.3)	23.2 (3.2)
Sex					
Female	20 (64.5%)	14 (70.0%)	14 (70.0%)	12 (66.7%)	13 (68.4%)
Male	11 (35.5%)	6 (30.0%)	6 (30.0%)	6 (33.3%)	6 (31.6%)
Race/ethnicity					
White	23 (74.2%)	18 (90.0%)	18 (90.0%)	16 (88.9%)	17 (89.5%)
South Asian	5 (16.1%)	1 (5.0%)	1 (5.0%)	1 (5.6%)	1 (5.3%)
Black	1 (3.2%)	1 (5.0%)	1 (5.0%)	1 (5.6%)	1 (5.3%)
Hispanic	1 (3.2%)	0 (0%)	0 (0%)	0 (0%)	0 (0%)
Southeast Asian	1 (3.2%)	0 (0%)	0 (0%)	0 (0%)	0 (0%)
Hair Colour					
Brown	11 (35.5%)	6 (30.0%)	6 (30.0%)	5 (27.8%)	6 (31.6%)
Blonde	10 (32.3%)	10 (50.0%)	10 (50.0%)	9 (60.0%)	9 (47.4%)
Black	8 (25.8%)	2 (10.0%)	2 (10.0%)	2 (11.1%)	2 (10.5%)
Red	2 (6.5%)	2 (10.0%)	2 (10.0%)	2 (11.1%)	2 (10.5%)
Handedness					
Right	27 (87.1%)	17 (85%)	17 (85%)	15 (83.3.6%)	16 (84.2%)
Left	3 (9.7%)	2 (10.0%)	2 (10.0%)	2 (11.1%)	2 (10.5%)
Ambidextrous	1 (3.2%)	1 (5.0%)	1 (5.0%)	1 (5.6%)	1 (5.3%)

**Table 2:** Mean (SD) taVNS amplitude in sham and active conditions.

Condition	Perception Threshold (mA)	Pain Threshold (mA)	Final Threshold (mA)
Sham	17.3 (3.5)	21.7 (4.8)	19.8 (4.1)
Active	19.9 (3.8)	22.3 (4.4)	20.8 (3.7)

Note. Values are presented as mean (SD) in milliamperes (mA). Perception threshold = the initial current at which taVNS stimulation was felt. Pain threshold = current just below the participant’s pain tolerance. Final threshold = taVNS stimulation level used for the task.

**Table 3:** Parcel-Wise statistical comparison of right motor tasks. Only parcels with an uncorrected *p* < 0.05 are reported. * represents FDR-corrected *p* < 0.05.

	Parcels	N	Baseline		Task		ΔHbO (µM)	*p*	FDR *p*	Cohen’s *d*
			*M*	*SD*	*M*	*SD*				
Right + Active	Left Inferior frontal gyrus, opercular part	26	0	0	0.033	0.066	0.033	0.017	0.071	0.50
	Left Paracentral Lobule	29	0	0	0.008	0.019	0.008	0.028	0.073	0.43
	Left Postcentral gyrus	26	0	0	0.048	0.065	0.048	0.001	0.012 *	0.74
	Left Precentral gyrus	28	0	0	0.050	0.078	0.050	0.002	0.013 *	0.64
	Left Supramarginal gyrus	15	0	0	0.019	0.029	0.019	0.022	0.071	0.67
Right + Sham	Left Inferior frontal gyrus, opercular part	26	0	0	0.042	0.078	0.042	0.010	0.033 *	0.55
	Right Inferior frontal gyrus, opercular part	26	0	0	0.025	0.058	0.025	0.033	0.072	0.44
	Left Paracentral Lobule	28	0	0	0.007	0.013	0.007	0.008	0.033 *	0.54
	Right Paracentral Lobule	15	0	0	0.006	0.009	0.006	0.015	0.038 *	0.72
	Left Postcentral gyrus	27	0	0	0.051	0.061	0.051	<0.001	0.002 *	0.84
	Left Precentral gyrus	27	0	0	0.055	0.084	0.055	0.002	0.013 *	0.66
	Left Supramarginal gyrus	14	0	0	0.014	0.023	0.014	0.039	0.073	0.61

**Table 4:** Parcel-wise statistical comparison of left motor tasks. Only parcels with an uncorrected *p* < 0.05 are reported. * represents *p* < 0.05.

Task	Parcels	N	Baseline		Task		ΔHbO (µM)	*p*	FDR *p*	Cohen’s *d*
			*M*	*SD*	*M*	*SD*				
Left + Active	Left Inferior frontal gyrus, opercular part	24	0	0	0.031	0.060	0.031	0.020	0.053	0.51
	Left Postcentral gyrus	25	0	0	0.029	0.042	0.029	0.002	0.011 *	0.69
	Right Postcentral gyrus	24	0	0	0.025	0.042	0.025	0.008	0.025 *	0.60
	Left Precentral gyrus	25	0	0	0.036	0.053	0.036	0.002	0.011 *	0.68
	Right Precentral gyrus	24	0	0	0.052	0.073	0.052	0.002	0.011 *	0.70
Left + Sham	Left Inferior frontal gyrus, opercular part	24	0	0	0.042	0.078	0.042	0.014	0.038 *	0.54
	Left Paracentral Lobule	26	0	0	0.004	0.009	0.004	0.034	0.063	0.44
	Left Postcentral gyrus	25	0	0	0.028	0.042	0.028	0.003	0.014 *	0.65
	Right Postcentral gyrus	24	0	0	0.028	0.040	0.028	0.003	0.014 *	0.69
	Left Precentral gyrus	25	0	0	0.036	0.057	0.036	0.004	0.014 *	0.63
	Right Precentral gyrus	24	0	0	0.056	0.076	0.056	0.001	0.014 *	0.74
	Right Supramarginal gyrus	18	0	0	0.010	0.017	0.010	0.023	0.049 *	0.59

## Data Availability

The raw data supporting the conclusions of this article can be made on reasonable request and at the discretion of the corresponding author.

## Supplementary Materials

The following supporting information can be downloaded at https://www.mdpi.com/article/10.3390/brainsci16020146/s1: Table S1. Mean number of channels that met pruning criteria across conditions for participants included in subsequent analyses. Table S2. AAL2 Parcel-wise t-stats between change in HbO concentration seen in active vs. Sham taVNS for the Right Motor task. Table S3. AAL2 Parcel-wise t-stats between change in HbR concentration seen in active vs. Sham taVNS for the Right Motor task. Table S4. AAL2 Parcel-wise t-stats between the change in HbO concentration seen in active vs. Sham taVNS for the Left Motor task. Table S5. AAL2 Parcel-wise t-stats between the change in HbR concentration seen in active vs. Sham taVNS for the Left Motor task. Table S6. Parcel-Wise Statistical Comparison of HbR in Right Motor Tasks. Only parcels with an uncorrected *p* < 0.05 are reported. Table S7. Parcel-Wise Statistical Comparison of HbR in Left Motor Tasks. Only parcels with an uncorrected *p* < 0.05 are reported. Figure S1. T-stat maps comparing HbR at baseline vs. task within conditions, and relative change in HbR concentration across right finger tapping tasks. For the uncorrected t-maps, only nodes with a *p* < 0.05 prior to FDR correction are shown. Figure S2. T-stat maps comparing HbR at baseline vs. task within conditions, and relative change in HbR concentration across left finger tapping tasks. For the uncorrected t-maps, only nodes with a *p* < 0.05 prior to FDR correction are shown.

## References

[1] (2024). GBD 2021 Stroke Risk Factor Collaborators. Global, regional, and national burden of stroke and its risk factors, 1990–2021: A systematic analysis for the Global Burden of Disease Study 2021. Lancet Neurol..

[2] Kwakkel G., Kollen B.J., van der Grond J., Prevo A.J. (2003). Probability of regaining dexterity in the flaccid upper limb: Impact of severity of paresis and time since onset in acute stroke. Stroke.

[3] Lang C.E., Strube M.J., Bland M.D., Waddell K.J., Cherry-Allen K.M., Nudo R.J., Dromerick A.W., Birkenmeier R.L. (2016). Dose response of task-specific upper limb training in people at least 6 months poststroke: A phase II, single-blind, randomized, controlled trial. Ann. Neurol..

[4] Daly J.J., McCabe J.P., Holcomb J., Monkiewicz M., Gansen J., Pundik S. (2019). Long-Dose Intensive Therapy Is Necessary for Strong, Clinically Significant, Upper Limb Functional Gains and Retained Gains in Severe/Moderate Chronic Stroke. Neurorehabil. Neural Repair.

[5] Dawson J., Engineer N.D., Cramer S.C., Wolf S.L., Ali R., O’Dell M.W., Pierce D., Prudente C.N., Redgrave J., Feng W. (2023). Vagus Nerve Stimulation Paired With Rehabilitation for Upper Limb Motor Impairment and Function After Chronic Ischemic Stroke: Subgroup Analysis of the Randomized, Blinded, Pivotal, VNS-REHAB Device Trial. Neurorehabil. Neural Repair.

[6] Bowles S., Hickman J., Peng X., Williamson W.R., Huang R., Washington K., Donegan D., Welle C.G. (2022). Vagus nerve stimulation drives selective circuit modulation through cholinergic reinforcement. Neuron.

[7] Baig S.S., Dorney S., Aziz M., Bell S.M., Ali A.N., Su L., Redgrave J.N., Majid A. (2025). Optimizing non-invasive vagus nerve stimulation for treatment in stroke. Neural Regen. Res. Dec..

[8] Baig S.S., Kamarova M., Bell S.M., Ali A.N., Su L., Dimairo M., Dawson J., Redgrave J.N., Majid A. (2023). tVNS in Stroke: A Narrative Review on the Current State and the Future. Stroke.

[9] Redgrave J.N., Moore L., Oyekunle T., Ebrahim M., Falidas K., Snowdon N., Ali A., Majid A. (2018). Transcutaneous Auricular Vagus Nerve Stimulation with Concurrent Upper Limb Repetitive Task Practice for Poststroke Motor Recovery: A Pilot Study. J. Stroke Cerebrovasc. Dis..

[10] Capone F., Miccinilli S., Pellegrino G., Zollo L., Simonetti D., Bressi F., Florio L., Ranieri F., Falato E., Di Santo A. (2017). Transcutaneous Vagus Nerve Stimulation Combined with Robotic Rehabilitation Improves Upper Limb Function after Stroke. Neural Plast..

[11] Baig S.S., Mooney C., McKendrick K., Duffy K.E.M., Ali A.N., Redgrave J.N., Herbert E., Waterhouse S., Su L., Drummond A. (2025). TRanscutaneous lImb reCovEry Post-Stroke (TRICEPS): Study protocol for a randomised, controlled, multiarm, multistage adaptive design trial. BMJ Open.

[12] Burger A.M., D’Agostini M., Verkuil B., Van Diest I. (2020). Moving beyond belief: A narrative review of potential biomarkers for transcutaneous vagus nerve stimulation. Psychophysiology.

[13] Rajiah R., Takahashi K., Aziz Q., Ruffle J.K. (2024). Brain effect of transcutaneous vagal nerve stimulation: A meta-analysis of neuroimaging evidence. Neurogastroenterol. Motil..

[14] Huo C., Xu G., Xie H., Chen T., Shao G., Wang J., Li W., Wang D., Li Z. (2024). Functional near-infrared spectroscopy in non-invasive neuromodulation. Neural Regen. Res..

[15] Vidal-Rosas E.E., von Luhmann A., Pinti P., Cooper R.J. (2023). Wearable, high-density fNIRS and diffuse optical tomography technologies: A perspective. Neurophotonics.

[16] Collins-Jones L.H., Gosse L.K., Blanco B., Bulgarelli C., Siddiqui M., Vidal-Rosas E.E., Duobaite N., Nixon-Hill R.W., Smith G., Skipper J. (2024). Whole-head high-density diffuse optical tomography to map infant audio-visual responses to social and non-social stimuli. Imaging Neurosci..

[17] Butt M.F., Albusoda A., Farmer A.D., Aziz Q. (2020). The anatomical basis for transcutaneous auricular vagus nerve stimulation. J. Anat..

[18] Frangos E., Ellrich J., Komisaruk B.R. (2015). Non-invasive Access to the Vagus Nerve Central Projections via Electrical Stimulation of the External Ear: fMRI Evidence in Humans. Brain Stimul..

[19] Huppert T.J., Diamond S.G., Franceschini M.A., Boas D.A. (2009). HomER: A review of time-series analysis methods for near-infrared spectroscopy of the brain. Appl. Opt..

[20] Tachtsidis I., Scholkmann F. (2016). False positives and false negatives in functional near-infrared spectroscopy: Issues, challenges, and the way forward. Neurophotonics.

[21] Butters E., Collins-jones L., Mesquita R., Acharya D., Mckiernan E., Laurell A.A.S., Low A., Srinivasan S., O’brien J., Su L. (2025). Brain Network Analysis in Alzheimer’s Disease and Mild Cognitive Impairment Using High-Density Diffuse Optical Tomography. J. Cereb. Blood Flow Metab..

[22] Wyser D., Mattille M., Wolf M., Lambercy O., Scholkmann F., Gassert R. (2020). Short-channel regression in functional near-infrared spectroscopy is more effective when considering heterogeneous scalp hemodynamics. Neurophotonics.

[23] Fiske A., de Klerk C., Lui K.Y.K., Collins-Jones L., Hendry A., Greenhalgh I., Hall A., Scerif G., Dvergsdal H., Holmboe K. (2022). The neural correlates of inhibitory control in 10-month-old infants: A functional near-infrared spectroscopy study. Neuroimage.

[24] Maintz J.B., Viergever M.A. (1998). A survey of medical image registration. Med. Image Anal..

[25] Rolls E.T., Joliot M., Tzourio-Mazoyer N. (2015). Implementation of a new parcellation of the orbitofrontal cortex in the automated anatomical labeling atlas. NeuroImage.

[26] Uchitel J., Blanco B., Vidal-Rosas E., Collins-Jones L., Cooper R.J. (2022). Reliability and similarity of resting state functional connectivity networks imaged using wearable, high-density diffuse optical tomography in the home setting. Neuroimage.

[27] Benjamini Y., Hochberg Y. (1995). Controlling the False Discovery Rate: A Practical and Powerful Approach to Multiple Testing. J. R. Stat. Soc. Ser. B Stat. Methodol..

[28] Turesky T.K., Turkeltaub P.E., Eden G.F. (2016). An Activation Likelihood Estimation Meta-Analysis Study of Simple Motor Movements in Older and Young Adults. Front. Aging Neurosci..

[29] Peng X., Baker-Vogel B., Sarhan M., Short E.B., Zhu W., Liu H., Kautz S., Badran B.W. (2023). Left or right ear? A neuroimaging study using combined taVNS/fMRI to understand the interaction between ear stimulation target and lesion location in chronic stroke. Brain Stimul..

[30] Wang L., Gao F., Dai Y., Wang Z., Liang F., Wu J., Wang M., Wang L. (2023). Transcutaneous auricular vagus nerve stimulation on upper limb motor function with stroke: A functional near-infrared spectroscopy pilot study. Front. Neurosci..

[31] Li S.Y., Xu K., Wang Y.X., Wang M.H., Li S.S., Lin F., Jiang Z.L. (2025). Task-specific cortical mechanisms of taVNS-paired task-oriented training for post-stroke upper extremity dysfunction under cognitive load: An fNIRS study. Front. Hum. Neurosci..

[32] Callaert D.V., Vercauteren K., Peeters R., Tam F., Graham S., Swinnen S.P., Sunaert S., Wenderoth N. (2011). Hemispheric asymmetries of motor versus nonmotor processes during (visuo)motor control. Hum. Brain Mapp..

[33] Srinivasan S., Acharya D., Butters E., Collins-Jones L., Mancini F., Bale G. (2024). Subject-specific information enhances spatial accuracy of high-density diffuse optical tomography. Front. Neuroergon..

[34] Kim A.Y., Marduy A., de Melo P.S., Gianlorenco A.C., Kim C.K., Choi H., Song J.J., Fregni F. (2022). Safety of transcutaneous auricular vagus nerve stimulation (taVNS): A systematic review and meta-analysis. Sci. Rep..

