# Supplementary material for: Transcutaneous Vagus Nerve Stimulation During Motor Activity in Healthy Volunteers: A High-Density Diffuse Optical Tomography Study
Source: Brain Sci. 2026 Jan 29;16(2):146. doi: 10.3390/brainsci16020146 (PMC12938713; doi:10.3390/brainsci16020146)
Supplement: Supplementary file 1 [file brainsci-16-00146-s001.zip › brainsci-4076726-supplementary.pdf]

Supplementary Material

Supplementary Table S1. *Mean number of channels that met pruning criteria across conditions for participants included in subsequent analyses.*

Condition	Right Motor + Sham taVNS	Right Motor + Active taVNS	Left Motor + Sham taVNS	Left Motor + Active taVNS
Mean Number of Good Channels	278.6 (64.9)	271 (64.4)	283.3 (65.3)	269.9 (64.6)

Supplementary Table S2. *AAL2 Parcel-wise t-stats between change in HbO concentration seen in active vs. Sham taVNS for the Right Motor task.*

Parcels	*N*	Change in Active		Change in Sham		Δ *Diff* (µM)	Uncorrected *p*	FDR *p*	Cohen's *d*
		M	*SD*	*M*	*SD*				
Frontal_Inf_Oper_L	27	0.035	0.066	0.045	0.078	-0.010	0.46	0.69	-0.14
Frontal_Inf_Oper_R	27	0.019	0.060	0.032	0.067	-0.013	0.28	0.69	-0.21
Paracentral_Lobule_L	29	0.008	0.019	0.007	0.013	0.002	0.48	.69	0.13
Paracentral_Lobule_R	16	0.003	0.012	0.005	0.009	-0.002	0.64	0.75	-0.12
Parietal_Inf_L	5	0.026	0.031	0.019	0.018	0.007	0.40	0.69	0.43
Postcentral_L	28	0.049	0.065	0.054	0.061	-0.005	0.45	0.69	-0.14
Postcentral_R	27	0.010	0.025	0.009	0.025	0.001	0.81	0.81	0.05
Precentral_L	28	0.053	0.078	0.057	0.082	-0.004	0.69	0.75	-0.08
Precentral_R	28	0.015	0.047	0.010	0.032	0.005	0.62	0.75	0.10
Supp_Motor_Area_L	8	0.013	0.019	0.001	0.009	0.011	0.14	0.69	0.58
Supp_Motor_Area_R	3	0.012	0.006	0.001	0.004	0.011	0.21	0.69	1.06
SupraMarginal_L	15	0.025	0.033	0.019	0.029	0.006	0.39	0.69	0.23
SupraMarginal_R	20	0.003	0.017	0.007	0.020	-0.004	0.33	0.69	-0.23

Supplementary Table S3. *AAL2 Parcel-wise t-stats between change in HbR concentration seen in active vs. Sham taVNS for the Right Motor task.*

Parcels	*N*	Change in Active		Change in Sham		Δ *Diff* (µM)	Uncorrected *p*	FDR *p*	Cohen's *d*
		M	*SD*	*M*	*SD*				
Frontal_Inf_Oper_L	27	-0.004	0.066	-0.023	0.062	0.018	0.19	0.45	0.26
Frontal_Inf_Oper_R	27	-0.014	0.038	-0.030	0.059	0.016	0.15	0.45	0.29
Paracentral_Lobule_L	29	-0.006	0.018	-0.004	0.021	-0.002	0.53	0.60	-0.12
Paracentral_Lobule_R	16	-0.004	0.010	-0.001	0.014	-0.003	0.51	0.60	-0.17
Parietal_Inf_L	5	-0.019	0.020	-0.014	0.014	-0.005	0.55	0.60	-0.29
Postcentral_L	28	-0.026	0.042	-0.031	0.046	0.005	.44	0.60	0.15
Postcentral_R	27	-0.006	0.015	-0.012	0.019	0.005	0.15	0.45	0.29
Precentral_L	28	-0.021	0.051	-0.030	0.063	0.009	0.34	0.60	0.18
Precentral_R	28	-0.004	0.030	-0.004	0.033	0.000	0.99	0.99	0.00
Supp_Motor_Area_L	8	-0.012	0.025	0.000	0.011	-0.012	0.17	0.45	-0.55
Supp_Motor_Area_R	3	-0.012	0.003	0.004	0.012	-0.016	0.21	0.45	-1.05
SupraMarginal_L	15	-0.015	0.030	-0.010	0.026	-0.005	0.48	0.60	-0.19
SupraMarginal_R	20	0.000	0.017	-0.008	0.018	0.008	0.09	0.45	0.39

Supplementary Table S4. *AAL2 Parcel-wise t-stats between the change in HbO concentration seen in active vs. Sham taVNS for the Left Motor task.*

Parcels	*N*	Change in Active		Change in Sham		Δ *Diff* (µM)	Uncorrected *p*	FDR *p*	Cohen's *d*
		M	*SD*	*M*	*SD*				
Frontal_Inf_Oper_L	24	0.031	0.060	0.042	0.078	-0.011	0.45	0.996	-0.16
Frontal_Inf_Oper_R	24	0.011	0.045	0.022	0.056	-0.012	0.40	0.996	-0.18

Paracentral_Lobule_L	26	0.007	0.019	0.004	0.009	0.003	0.40	0.996	0.17
Paracentral_Lobule_R	15	0.009	0.017	0.009	0.018	0.000	0.98	0.996	0.01
Parietal_Inf_L	6	0.006	0.019	0.007	0.009	-0.001	0.90	0.996	-0.05
Postcentral_L	25	0.029	0.042	0.028	0.042	0.002	0.82	0.996	0.05
Postcentral_R	24	0.025	0.042	0.028	0.040	-0.002	0.67	0.996	-0.09
Precentral_L	25	0.036	0.053	0.036	0.057	0.000	0.996	0.996	0.00
Precentral_R	24	0.052	0.073	0.056	0.076	-0.005	0.48	0.996	-0.15
Supp_Motor_Area_L	8	0.006	0.016	0.013	0.017	-0.007	0.06	0.719	-0.81
Supp_Motor_Area_R	3	-0.002	0.003	0.006	0.008	-0.008	0.28	0.996	-0.84
SupraMarginal_L	14	0.010	0.018	0.010	0.021	0.000	0.99	0.996	0.00
SupraMarginal_R	18	0.012	0.025	0.010	0.017	0.002	0.74	0.996	0.08

Supplementary Table S5. *AAL2 Parcel-wise t-stats between the change in HbR concentration seen in active vs. Sham taVNS for the Left Motor task.*

Parcels	*N*	Change in Active		Change in Sham		Δ *Diff* (µM)	Uncorrected *p*	FDR *p*	Cohen's *d*
		M	*SD*	*M*	*SD*				
Frontal_Inf_Oper_L	24	-0.009	0.049	-0.008	0.042	-0.001	0.91	0.91	-0.02
Frontal_Inf_Oper_R	24	-0.017	0.035	-0.003	0.049	-0.014	0.25	0.62	-0.24
Paracentral_Lobule_L	26	-0.004	0.018	0.000	0.009	-0.004	0.27	0.62	-0.22
Paracentral_Lobule_R	15	-0.009	0.011	-0.006	0.015	-0.002	0.56	0.66	-0.15
Parietal_Inf_L	6	-0.006	0.013	-0.009	0.016	0.003	0.53	0.66	0.28
Postcentral_L	25	-0.013	0.027	-0.004	0.023	-0.009	0.16	0.62	-0.29
Postcentral_R	24	-0.016	0.032	-0.012	0.029	-0.004	0.48	0.66	-0.15

Precentral_L	25	-0.008	0.033	-0.006	0.037	-0.002	0.85	0.91	-0.04
Precentral_R	24	-0.034	0.056	-0.024	0.048	-0.010	0.21	0.62	-0.26
Supp_Motor_Area_L	8	0.002	0.027	-0.005	0.024	0.007	0.49	0.66	0.26
Supp_Motor_Area_R	3	0.005	0.011	0.000	0.003	0.004	0.44	0.66	0.55
SupraMarginal_L	14	-0.009	0.017	-0.003	0.018	-0.006	0.25	0.62	-0.32
SupraMarginal_R	18	-0.009	0.024	-0.003	0.014	-0.007	0.29	0.62	-0.26

Supplementary Table S6. *Parcel-Wise Statistical Comparison of HbR in Right Motor Tasks. Only parcels with an uncorrected p < 0.05 are reported. * represents p < 0.05.*

	Parcels	N	Baseline		Task		Δ HbR (μM)	*p*	FDR *p*	Cohen's *d*
			M	*SD*	*M*	*SD*				
Right + Active	Left Postcentral Gyrus	26	0	0	-0.023	0.038	-0.023	0.004	0.06	-0.61
	Left Supplementary Motor Area	3	0	0	-0.012	0.003	-0.012	0.03	0.17	-3.48
Right + Sham	Left Inferior frontal gyrus, opercular part	26	0	0	-0.024	0.052	-0.024	0.03	0.11	-0.47
	Right Postcentral gyrus	27	0	0	-0.028	0.042	-0.028	0.002	0.03 *	-0.66
	Left Postcentral gyrus	26	0	0	-0.010	0.017	-0.010	0.01	0.03 *	-0.60
	Left Precentral gyrus	27	0	0	-0.026	0.060	-0.026	0.03	0.11	-0.43

Supplementary Table S7. *Parcel-Wise Statistical Comparison of HbR in Left Motor Tasks. Only parcels with an uncorrected p < 0.05 are reported. * represents p < 0.05.*

Task	Parcels	N	Baseline		Task		Δ HbR (μM)	*p*	FDR *p*	Cohen's *d*
			M	*SD*	*M*	*SD*				
Left + Active	Left Inferior frontal gyrus, opercular part	24	0	0	-0.017	0.035	-0.017	0.03	0.07	-0.48

	Left Paracentral Lobule	15	0	0	-0.009	0.011	-0.009	0.01	0.07	-0.76
	Left Postcentral gyrus	25	0	0	-0.013	0.027	-0.013	0.03	0.07	-0.47
	Right Postcentral gyrus	24	0	0	-0.016	0.032	-0.016	0.02	0.07	-0.49
	Right Precentral gyrus	24	0	0	-0.034	0.056	-0.034	0.01	0.07	-0.59
Left + Sham	Right Precentral gyrus	24	0	0	-0.024	0.048	-0.024	0.03	0.33	-0.49

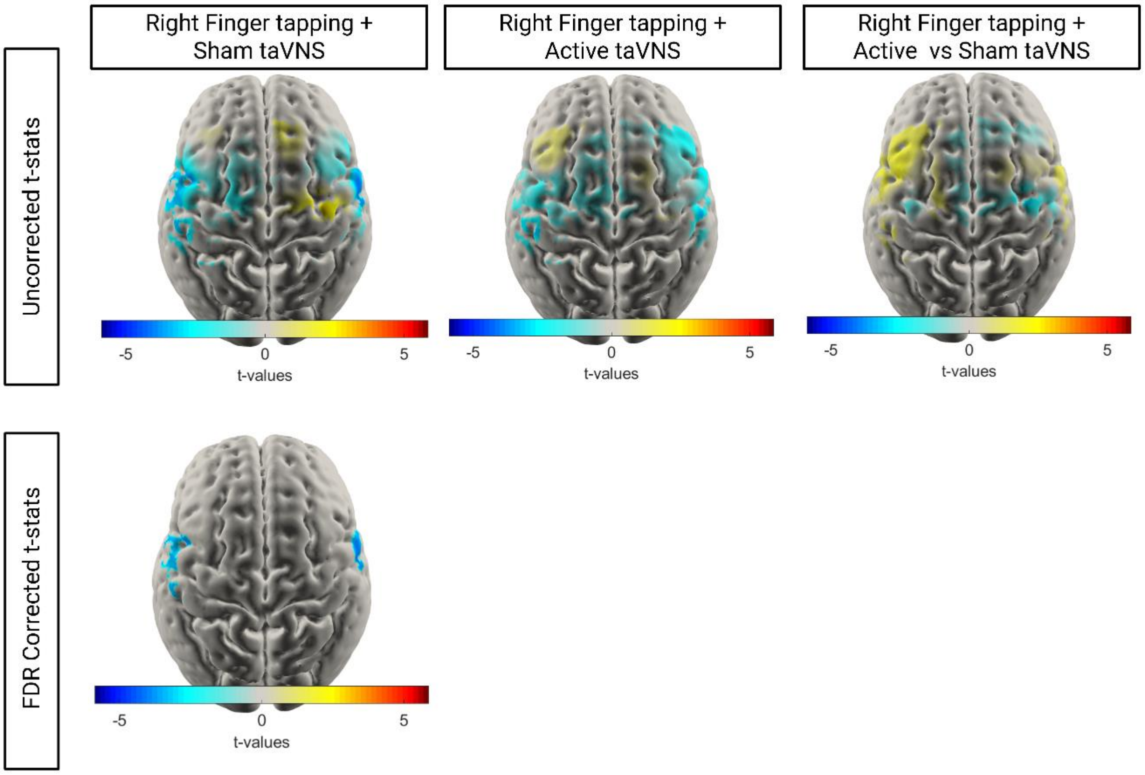

Supplementary Figure S1. *T-stat maps comparing HbR at baseline vs task within conditions, and relative change in HbR concentration across right finger tapping tasks. For the uncorrected t-maps, only nodes with a p < 0.05 prior to FDR correction are shown.*

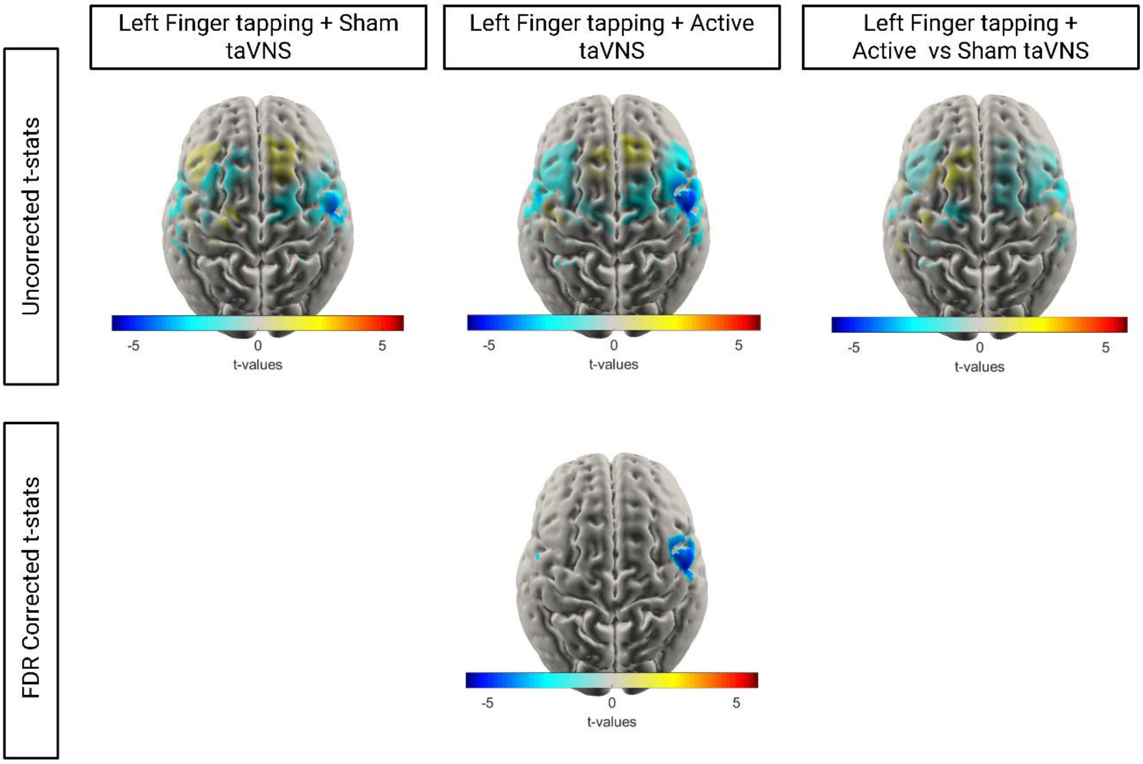

Supplementary Figure S2. *T-stat maps comparing HbR at baseline vs task within conditions, and relative change in HbR concentration across left finger tapping tasks. For the uncorrected t-maps, only nodes with a p < .05 prior to FDR correction are shown.*
